# Characterisation of *Tenebrio molitor* Reared on Substrates Supplemented with Chestnut Shell

**DOI:** 10.3390/insects15070512

**Published:** 2024-07-09

**Authors:** Irene Ferri, Matteo Dell’Anno, Mattia Spano, Benedetta Canala, Beatrice Petrali, Matilda Dametti, Stefano Magnaghi, Luciana Rossi

**Affiliations:** 1 Department of Veterinary Medicine and Animal Sciences—DIVAS, University of Milan, 26900 Lodi, Italy; irene.ferri@unimi.it (I.F.); matteo.dellanno@unimi.it (M.D.); benedetta.canala@unimi.it (B.C.); beatrice.petrali@studenti.unimi.it (B.P.); mati.dametti@gmail.com (M.D.); 2 Food Chemistry Lab, Department of Chemistry and Technology of Drugs, Sapienza University of Rome, 00185 Rome, Italy; mattia.spano@uniroma1.it; 3 Italian Cricket Farm, 10060 Scalenghe, Italy; ricerca@italiancricketfarm.com

**Keywords:** mealworm, by-products, antioxidant, antimicrobial, *E. coli* O138, innovative substrates, *Castanea sativa*, amino acids, pyroglutamate, asparagine

## Abstract

**Simple Summary:**

Due to the growing world population, the sustainability of food and feed sources with high nutritional value has become a crucial issue. In this scenario, insects can constitute a low-impact protein source with high nutritional value. The growth and nutrient composition of insects are potentially influenced by rearing conditions, particularly by the selected growth substrate. In this study, we evaluated the chemical and functional characteristics of *Tenebrio molitor* larvae reared on different growth substrates: a traditional wheat bran substrate and an innovative substrate consisting of wheat bran supplemented with chestnut shell, a by-product of the chestnut agro-industrial chain. The results showed that the innovative growth substrates positively influenced the insects’ survival suggesting a beneficial effect on larval health. The enrichment of the growth substrate with chestnut shell modified the protein and amino acid profile of insect meals, possibly indicating a shift in their metabolism. In addition, insect meals obtained from larvae reared on chestnut-shell-enriched substrate exhibited higher antibacterial and antioxidant activity, suggesting a potential beneficial effect when included in animal feed. Our results showed positive outcomes related to the design of innovative strategies for insect rearing, enriching larvae meal with beneficial health properties in line with sustainability and One Health principles.

**Abstract:**

*Tenebrio molitor* larvae represent a sustainable protein source for food and feed. The aim of this study was to evaluate the supplementation of chestnut shell, a by-product of the agro-industrial chain, in growth substrates for *T. molitor* larvae rearing. Seven-week-old larvae were reared on three different growth substrates: the control group (CTRL) was fed wheat bran, treatment group one was fed wheat bran supplemented with 12.5% *w*/*w* chestnut shell (TRT1), and treatment group two was fed wheat bran supplemented with 25% *w*/*w* chestnut shell (TRT2). Larval weight, substrate consumption, and mortality were recorded weekly. After 14 days, insect meals were produced for bromatological and colorimetric analysis, and bacterial inhibition activity assay using a microdilution method. The amino acid profile of insects was determined using quantitative nuclear magnetic resonance spectroscopy. Our results showed a lower feed conversion ratio and higher larval survival rate % in TRT2 compared to CTRL (*p* < 0.05). Proteins and lipids of TRT2 were higher than other groups (*p* < 0.05). Important differences were observed in the amino acid profile of TRT1 and TRT2 compared to CTRL (*p* < 0.05). TRT1 and TRT2 showed higher *E. coli* inhibitory activity than CTRL (*p* < 0.05). In conclusion, chestnut shell supplementation improved the survival and functional characteristics of larvae and likely impacted the insects’ metabolism.

## 1. Introduction

Insects are sustainable food sources with a high concentration of proteins and fat, as well as an appreciable content of micronutrients that could support antioxidative and antimicrobial defence (tocopherol and polyphenols) [1,2,3]. In comparison to traditional protein sources like soybean and fishmeal, insect meals offer optimised utilisation of inputs related to the product quality while also considering their environmental footprint [4,5,6]. Specifically, insects possess the ability to transform low-value by-products into high-quality ingredients [7]. Among the edible insects, the first species to receive a positive opinion from the European Food Safety Authority (EFSA) as a Novel Food was *Tenebrio molitor* (Regulation EU 2015/2283). This insect is a yellow mealworm with four stages of development, i.e., egg, larva, pupa, and adult. *T. molitor*’s chemical composition suggests that among these stages, the larval phase is the most interesting from the nutritional point of view. In addition to the development phase, the growth substrate affects the final composition of insect meals influencing, in particular, the growth curve of larvae as well as their protein and fat content [8,9]. In this regard, mealworms successfully convert organic waste into proteins suitable for animal nutrition. By using waste as growth substrate, it is possible not only to valorise organic waste but also to promote environmental sustainability. This approach allows *T. molitor* to be more competitive with traditional protein sources.

In recent years, several studies have tested the use of agro-industrial by-products as growth substrates for *T. molitor* with sometimes contrasting results, depending on the percentage of inclusion of the by-product with the traditional substrate (e.g., wheat bran). For example, citrus residues have shown promising results in terms of larval growth rates, as has the use of tomato pomace, which has recorded higher substrate consumption by insects [10]. Conversely, it has been observed that the combination of bran and tomato residue did not increase larval growth and, on the contrary, revealed high mortality levels. Nevertheless, several studies report the accumulation of bioactive compounds, such as flavonoids in the case of potato peel administration, demonstrating that the use of by-products can positively influence functional capacities through the transfer of bioactive molecules [11,12].

Insects have been recognised not only as a potential source of protein for human and animal consumption but also for their functional properties. In vitro studies have shown that *T. molitor* larvae exhibit bacterial inhibition activity induced by bioactive molecules, i.e., chitin, lauric acid and antimicrobial peptides [13]. Chitin, a component of the insect’s exoskeleton, has been observed to have prebiotic and antimicrobial activity against pathogens under certain circumstances thus possibly contributing to animal health [14]. Lauric acid, a fatty acid particularly abundant in some insect species, exhibits antimicrobial properties when administered in animal feed [15,16]. Furthermore, insects produce antimicrobial peptides (AMP) with broad-spectrum activity against Gram-positive, and Gram-negative bacteria, protozoa, viruses, and fungi [17]. To date, several in vivo studies have already demonstrated positive effects correlated with insect-based feed formulas for pigs and poultry with encouraging results on growth performance and animal health [18]. Nevertheless, no study has investigated the administration of larvae reared on substrates enriched with functional ingredients. Chestnut (*Castanea sativa*) production has significantly increased in recent years due to the discovery of health benefits provided by their bioactive molecules [19]. This increased demand has resulted in a relevant growth in chestnut agro-industrial residues such as chestnut shells, burrs, and wood. Although chestnut shells are considered by-products, the pericarp (outer shell 8.9–13.5%) and tegument (inner shell 6.3–10.1%) are valuable sources of phenols. These plant compounds are known for their antioxidants and antimicrobial effects [20]. Previous in vivo and in vitro studies have already explored the prospective use of chestnut extracts in animal nutrition, discovering several beneficial properties: antioxidant activity and heavy metals chelation, as well a positive effect on gut health [21,22,23].

However, there are no studies in the literature that investigated the effects of chestnut by-products in rearing substrates of larvae. For this reason, the aim of this study was to evaluate the effects of chestnut shell supplementation in growth substrates on insect performance and the nutritional and functional characteristics of *T. molitor* meals.

## 2. Materials and Methods

### 2.1. Experimental Design

**Figure 1:**
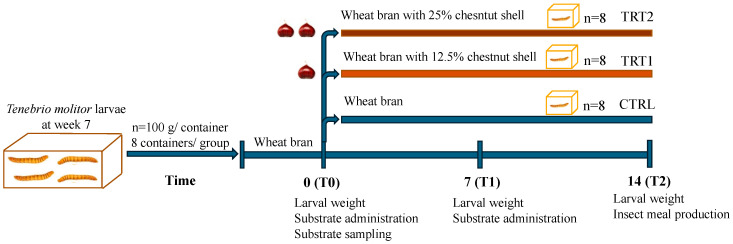
Experimental design.

**Figure 2:**
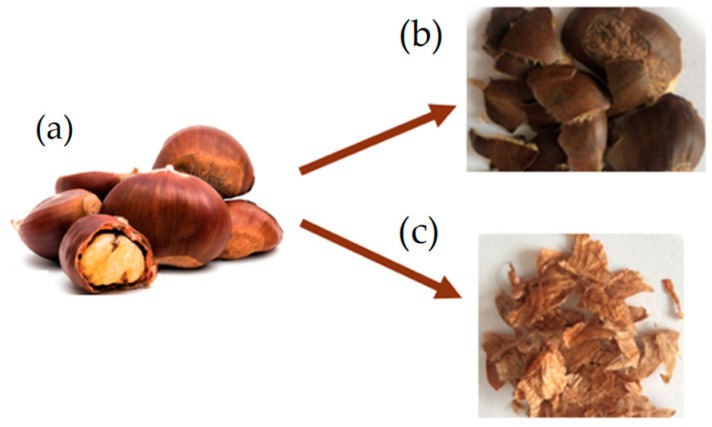
Chestnut fruit (**a**) with pericarp (**b**) and integument (**c**).

In this study, 7 week-old larvae of *Tenebrio molitor* grown on wheat bran substrate and vegetables (potato and carrots) as a source of hydration were used. For the experiment (Figure 1), a total of 2.4 kg of *T. molitor* larvae were randomly allocated among 24 plastic containers (27 × 39 × 14 cm), with each container holding 100 g of larvae (approximately corresponding to 500 larvae). Larvae were divided into three groups (8 replicates/group) according to the different growth substrates. The control group (CTRL, *n* = 8) was reared on wheat bran, treatment group 1 (TRT1, *n* = 8) received wheat bran supplemented with 12.5% *w*/*w* chestnut shell, and treatment group 2 (TRT2, *n* = 8) received wheat bran supplemented with 25% *w*/*w* chestnut shell. Wheat bran (dry matter: 85%; ash: 8%; fibre: 10%; crude protein: 14%) was supplied by a local farmer whereas chestnut shell (dry matter: 93.39%; ash: 1.63%; crude protein: 3.24%; fibre: 14.9%; lipids: 0.8%; carbohydrates: 78.3%) was provided by the Luciniera Farm (Modena, Italy) as a waste product of the agro-industrial supply chain of chestnut production. Chestnut shell, the skin of chestnut fruit (Figure 2a), is composed of two layers: pericarp and integument (Figure 2b,c). Both were used for the current study. Before the experimental trial, the product was subjected to a drying process at 65 °C for 48 h and ground through a mill with a sieve of 0.5 mm pore diameter.

All groups received sprayed water at days 0, 2, 4, 6, 8, 10 and 12 (10 mL/day). The rearing experiment lasted 14 days to obtain fully grown mealworms i.e., edible insects at the last period of the larval phase before becoming pupae. The development of larvae was monitored daily maintaining larvae under controlled conditions (26 ± 2 °C, 60–75% relative humidity). The study was carried out in the insect rearing facility of the Italian Cricket Farm s.r.l. (Pinerolo, Italy).

Growth substrates were collected from each replicate, and fresh substrates were provided weekly according to the respective treatment to maintain a larvae-to-substrate ratio of 2:1. Samples of residual growth substrate were collected at days 7 and 14 and stored at −20° for further analysis. The weight of larvae per container was registered every 7 days. At the end of the experimental trial (day 14), larvae were separated from the substrate using a mesh sieve (ø 300 µm) [24] and total insect biomass was weighed on a scale with an accuracy of 0.01 g (B2002-S, Mettler Toledo, Milan, Italy). After 24 h of starvation, the total larvae of each container were cooked by drying with a microwave (model CMG2071M, Candy Hoover Group S.r.l., Brugherio, Italy), maintaining a maximum input power of 120 W with a frequency of 2450 MHz for 5 min [25]. After that, dead larvae were used to obtain insect meals through a flour mill, resulting in 8 replicates per group, and analysed in the laboratory.

### 2.2. Growth Performance and Feed Conversion Ratio

The weight of residual substrates in grams was collected after separation from the larvae at days 7 and 14. Using a mesh sieve (square mesh ø 150 μm), substrates were divided into faeces and non-ingested feed. The difference between the total administered substrate and the residual diet was used to calculate the feed consumption (%).

The feed conversion ratio (FCR) was used to assess the feed conversion efficiency on a dry matter basis. It was calculated by dividing the weight of the ingested feed by the weight gained.

The number of dead larvae was registered weekly, along with the separation of the insects and residual substrates. The survival rate was calculated as the percentage of live larvae at the end of the trial. Specifically, the initial number of larvae was estimated based on the average weight of *T. molitor* larvae at the age of 7 weeks (~0.2 g) concerning the total weight of each container, and the weight of dead larvae was subtracted from the recorded weight after 7 and 14 days. The survival rate was then calculated based on the number of live larvae at 14 days in relation to the total number of larvae at the beginning of the experimental period.

### 2.3. Chemical Characterisation of Rearing Substrate

The chemical composition of growth substrates, administered on day 0 and collected on day 14, was assessed post-milling through a 1 mm screen grid according to the “Official Methods of Analysis” [26]. Dry matter was measured by placing samples in pre-weighed aluminium bags and subsequently dried in a forced-air oven at 65 °C for 24 h (AOAC method 930.15). Lipid content (ether extract, EE) was evaluated using ethyl ether in a Soxtec extractor (AOAC 2003.05). Total ash content was obtained after incineration at 550 °C for 3 h (AOAC method 942.05). Crude proteins (CP) were determined using a Kjeldahl system with 6.25 as the average nitrogen conversion coefficient for vegetable growth substrates (AOAC method 2001.11). Crude fibre (CF) was determined using the official AOACS Ba 6a-05 method employing filtering bags. Non-structural carbohydrates (NSC) were obtained by subtracting all assessed nutrients from 100, i.e., [100 − (moisture% + ash% + EE% + CP% + CF%)]. All batches of rearing substrates were analysed separately, after proper homogenisation to ensure sample representativity, as independent replicates for each group (*n* = 8 replicates). Each replicate was analysed in technical triplicate, repeating the analysis procedure three times for each batch of samples (24 total determinations).

### 2.4. Chemical Characterisation and Colour Analysis of Tenebrio molitor Larvae Meal

The chemical composition of *T. molitor* meal was evaluated following the analysis described in the previous section, except for CP. To determine protein content, a nitrogen-to-protein conversion factor of 4.76 was used, excluding the nitrogen from chitin as suggested by Jansen et al. [27]. All batches of insect meals were analysed separately as independent replicates for each group (*n* = 8).

An analysis of insect meal colour was performed to measure the pigmentation of larvae, which is indicative of functional molecules [28]. Utilising a Minolta Croma-Meter CR-400 colorimeter (Minolta Camera Co., Ltd., Osaka, Japan), the colour values were obtained. Data acquisition involved direct contact between the colorimeter’s sensing head and the samples. The characterisation of insect meals was analysed using three parameters: L* (a lightness scale from 0 to 100 where 0 represents black and 100 represents white), a* (measuring greenness/redness scale) and b* (indicating blueness/yellowness scale). The colour difference (ΔE) between two samples was calculated using the following formula:

$$\Delta E=\sqrt{{\left(\Delta L*\right)}^{2}+{\left(\Delta a*\right)}^{2}+{\left(\Delta b*\right)}^{2}}$$

If ΔE falls between 1 and 2, it implies that only an experienced observer can detect the difference [29].

All measurements were conducted in triplicate for each sample.

### 2.5. In Vitro Digestion of Insect Meals

Digestion was performed according to the method adopted by Reggi et al. [30] with slight adjustments. Specifically, 1 g of each insect meal was mixed with 20 mL of distilled H_2_O and incubated for 5 min at 37 °C under stirring. After that, the digestion procedure involved three phases. For the oral phase, 150 mg of α-amylase (A3176, Sigma-Aldrich, St. Louis, MO, USA) in 1 mL of 1 mM CaCl_2_ (pH 7) was added to the samples which were then incubated for 30 min at 37 °C on a shaker. For the gastric phase, the pH was decreased to 2 with HCl (6 M) and 100 mg of pepsin (P7000, Sigma-Aldrich, St. Louis, MO, USA) was added in 2 mL of HCl (0.1 M). The samples were then incubated for 120 min at 37 °C on a shaker. For the small intestinal phase, the pH was increased to 7 with NaOH (6 M) and 200 mg of pancreatin (P1750, Sigma-Aldrich, St. Louis, MO, USA) and 50 mg of bile (B8631, Sigma-Aldrich, St. Louis, MO, USA) in 2 mL of NaHCO_3_ 0.5 M were added to the samples before carrying out the final incubation of 180 min at 37 °C on a shaker.

At the end of digestion, the in vitro samples digested were centrifugated and the supernatant was filtered using pre-weighted Whatman filter paper (55 mm, grade 54, Cytiva, WA, USA). The filters containing the undigested portion were dried for three hours later. At the end of the three hours, the filters were weighed again to determine the weight of the digested meal.

### 2.6. Nuclear Magnetic Resonance (NMR) Spectroscopy of Free Amino Acid Profile

For the NMR analysis of free amino acid content, the Bligh-Dyer extract protocol was applied. Specifically, 100 mg of each sample was added to 3 mL of CH_3_OH/CHCl_3_ 2:1 *v*/*v* solution and 0.8 mL of distilled water. After sonication, a further 1 mL of CHCl_3_ and 1 mL of H_2_O were added, obtaining a two-phase system (hydroalcoholic and organic). The system was then centrifugated and the hydroalcoholic phase was isolated. The same procedure was applied two more times on the residual pellet to achieve a quantitative extraction. Finally, the reunited hydroalcoholic phases were dried under N_2_ flux. Sample preparation for NMR analysis was carried out by solubilising the dried extract in 1 mL of 100 mM phosphate buffer/D_2_O, containing 0.5 mM TSP (3-(trimethylsilyl)propionic acid sodium salt) as an internal standard and transferring 700 µL of this solution into a 5 mm NMR tube.

Analyses were carried out using a 600 MHz spectrometer (Jeol JNM-ECZ 600 R) equipped with a 5 mm probe (FG/RO DIGITAL AUTOTUNE). H NMR experiments were conducted using the same acquisition and processing parameters previously reported for the analysis of edible insects in the same conditions [31]. The ^1^H NMR amino acid signals used for integration and quantification are reported in Table 1.

### 2.7. Bacterial Growth Inhibitory Activity of Insect Meal Extracts

Methanol and deionised water were used to obtain extracts of dried *T. molitor* larvae meals from the CTRL, TRT1 and TRT2 groups [32]. In detail, a solution of 1.5 mL of methanol and deionised water (50:50, *v*/*v*) was used to dilute 225 mg of insect meals, chosen randomly from replicates (*n* = 8/group). Each replicate was analysed in technical triplicate, repeating the analysis procedure three times for each batch of samples, resulting in a total of 24 determinations. After that, the mixture was vortexed and left to be stirred at room temperature (30 min). Samples were centrifuged for 15 min at 10,000 rpm at 4 °C and following this, supernatants were collected and filtered using a syringe filter (0.45 μm). Each sample from a single replicate was extracted three times, and the resultant supernatants were combined in a single tube and then stored at −20 °C. Once the insect meals were extracted, bacterial growth inhibition was evaluated. The antibacterial activity of insect meals was performed using the *Escherichia coli* O138 strain as a representative model for gastrointestinal disorders, provided by the strain collection at the Department of Veterinary Medicine and Animal Sciences, University of Milan. The strain was genetically characterised for genes encoding two virulence factors: adhesive fimbria F18 and verocytotoxin 2e (VT2e) [30]. A liquid culture growth inhibition assay was performed using *E. coli* O138 to evaluate the ability of the bioactive compound extracts to inhibit bacterial growth. For the experiment, an overnight culture of *E. coli* O138 in Luria-Bertani (LB) broth was used as the inoculum.

The growth inhibition assay proceeded as follows: extracts were diluted in LB liquid medium at concentrations of 1:4 to avoid the methanol used for the extraction interfering with the bacterial growth. A 96-well plate was set up with a total of 100 μL of the diluted extract and 30 μL of *E. coli* culture diluted at OD600 = 0.05 ± 0.02 inoculum was added. Positive controls were prepared by adding 30 μL of *E. coli* inoculum to methanol/deionised water solution (50:50, *v*/*v*) diluted 1:4 to assess bacterial growth in the extraction solvent without insect extract. To correct for the background colour, 30 μL of LB and LB with extract without *E. coli* inoculum as negative control was included. Samples were then incubated at 37 °C in a shaking incubator for six hours. After this time, the growth rate of *E. coli* was estimated hourly for six hours (T0, T1, T2, T3, T4, T5, and T6) by measuring the absorbance with a microplate spectrophotometer at an optical density (OD) of 620 nm. The measured OD was converted to log_10_ of the number of cells/mL, considering 1 OD = 1 × 10^9^ cells/mL [33].

### 2.8. Statistical Analysis

All data were analysed using GraphPad Prism statistical software (Version 9.1.1). Data on insect growth and microbial growth inhibition were analysed using a generalised linear model after evaluating the Q-Q plots and performing tests for normality and homoscedasticity (Shapiro–Wilk test and Bartlett’s test, respectively). The model included the fixed effects of treatment and time (day or hour) and their interaction. Chemical composition and amino acid content, except pyroglutamate, were analysed using one-way analysis of variance (ANOVA) after a statistical test for normality (Shapiro–Wilk test) and homoscedasticity (Bartlett’s test). Student’s *t*-test was used to analyse pyroglutamate values because only a comparison between TRT1 and TRT2 was possible. The Kruskal–Wallis test was performed for the analysis of colorimetric data and other results that did not fit the normal distribution. The multivariate analysis of principal component analysis (PCA) was used to analyse the data on the free amino acid mineral profiles of different larvae meals. Mortality was analysed by calculating the mean of the number of dead larvae recorded during the 14 day test period and comparing group means using the ANOVA for unpaired samples. Post hoc Sidak’s or Mann–Whitney’s tests were performed to separate means. Data on growth performance, microbial growth inhibition, feed conversion efficiency, and mortality were presented as mean ± standard error. Differences were considered statistically significant for *p* < 0.05.

## 3. Results

### 3.1. Effect of Growth Substrates on Insect Growth Rate and Feed Conversion Ratio

**Figure 3:**
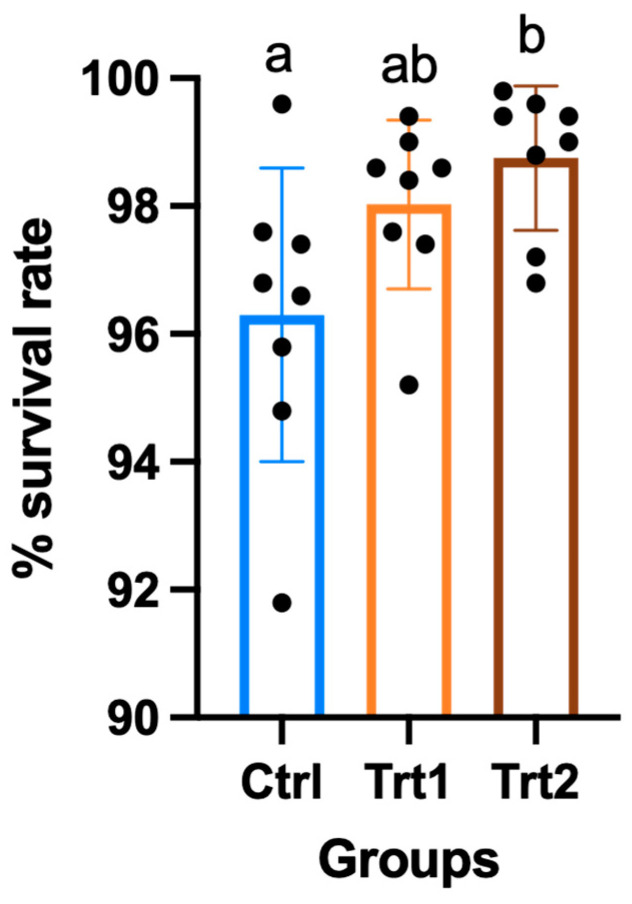
Percentage of survival of larvae in the control (CTRL) and treatment groups that received a supplementation of 12.5% chestnut shell (TRT1) and 25% chestnut shell (TRT2) over 14 days of the trial. All values are presented as ± standard deviations. Lowercase letters indicate statistically significant differences among tested groups (ANOVA, *p* = 0.0222; F = 4.59; DFn = 2, DFd = 21).

The growth performance of insects reared on different growth substrates showed significant differences except for the average weight (Table 2). After seven days of the trial, the CTRL group had consumed a higher quantity of substrate compared to TRT1 (*p* = 0.0329) and TRT2 (*p* = 0.0179) and from 7 to 14 days, the substrate consumption was similar. Despite a slight difference during the first week of the experiment, *T. molitor* larvae showed no differences in average weights. At day 14, the weight increased with data equal to 104.9 g for CTRL (mean of n larvae/cassette at the end of the trial = 482 ± 11), 109.4 g for TRT1 (mean of n larvae/cassette at the end of the trial = 490 ± 7) and 112.3 g for TRT2 (mean of n larvae/cassette at the end of the trial = 494 ± 6) with no significant differences among the groups. Comparable results were obtained for growth rate % in the CTRL, TRT1 and TRT2 after 7 days from the beginning of the trial; whereas, at the end of the experiment, the growth rate in the CTRL group was significantly lower than in TRT2 (*p* = 0.0253). The growth rate % of TRT1 did not show significant differences from other groups, although it tended to be higher than CTRL. The FCR of CTRL was different from TRT1 (*p* = 0.0071) and TRT2 after 7 days from the beginning of the trial, showing the lowest FCR in the CTRL group *(p* = 0.0217). At the end of the trial, the CTRL (11.79) group registered the highest FCR with significant differences compared to TRT2 (*p* = 0.0415) but it was statistically similar to TRT1. The TRT2 group registered the highest survival rates compared to the TRT1 and CTRL groups (*p* < 0.05; Figure 3).

### 3.2. Chemical Composition of Rearing Substrates

The experimental substrates showed no significant differences in main nutrients except for the protein content (Table 3). A different trend was registered for the proteins administered to the different groups with a higher protein content in the CTRL than the TRT1 and TRT2 groups with values of 17.00%, 14.48% and 15.09%, respectively, on a dry matter basis (*p* = 0.0001).

### 3.3. Chemical Composition, Digestibility and Colorimetric Analysis of Tenebrio molitor Larvae Meal

The chemical composition of the *T. molitor* larvae meal obtained from the experimental groups showed significant differences in the content of principal nutrients (Table 4). Specifically, the CTRL group produced insect meals with lower humidity compared to the treatment groups (*p* = 0.0002). Proteins of insect meals showed significant differences with higher content registered in the TRT1 (51.96%) compared to the CTRL (44.52%) and TRT2 (46.22%) groups (*p* = 0.0391). TRT1 also produced insect meals richer in lipids compared to the TRT2 and CTRL groups (*p* = 0.0123). On the contrary, ash in TRT1 was lower than in CTRL and TRT2 with significant differences between the two treatments (*p* < 0.05).

Insect meal digestibility values were 61.24 ± 3.12%, 60.07 ± 8.30% and 57.11 ± 3.12%, respectively, for the CTRL, TRT1 and TRT2 groups with no significant differences (ANOVA, *p* = 0.6506; F = 0.46; DFn = 2, DFd = 6).

L*, a* and b* parameters of the CTRL, TRT1, and TR2 insect meals were evaluated using a colorimeter (Table 5). After that, the colour variation was expressed as ΔE, which was equal to 5.59 between CTRL and TRT1, and 3.35 between CTRL and TRT2, respectively. Insects reared on innovative substrates showed a slightly lower value of L* compared to the CTRL group, indicating a darker colour of meals. Specifically, L* differed significantly between the CTRL and TR1 groups (*p* = 0.0014), whereas comparable results were observed for a* index between the groups. Moreover, samples from the CTRL group registered a higher b* index than the group fed with 12.5% chestnut shells (*p* = 0.0080).

### 3.4. Free Amino Acid Profiles of Insect Meals

**Figure 4:**
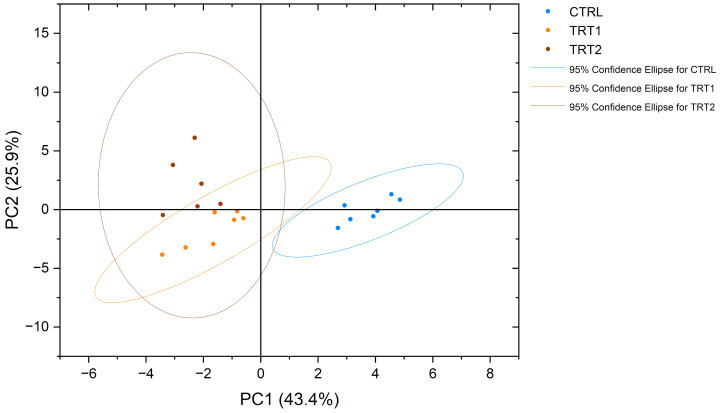
Principal component analysis (PCA) of free amino acid profiles of *Tenebrio molitor* meals of different growth substrates: wheat bran (CTRL), wheat bran with 12.5 and 25% chestnut shell (TRT1 and TRT2).

NMR spectroscopy made it possible to obtain a complete view of the free amino acid profiles of the analysed samples. Interestingly, eighteen proteinogenic amino acids and pyroglutamate, an amino acid derivative, were detected and measured. The obtained profiles showed significant differences among the experimental groups (Table 6). From a qualitative point of view, asparagine (Asn) was detected only in the CTRL group and not in TRT1 and TRT2, whereas the opposite trend was observed for pyroglutamate (PyroGlu). Among all groups, the most abundant free amino acids in *T. molitor* were proline (Pro), arginine (Arg) and tyrosine (Tyr). In contrast, methionine (Met) was the least abundant amino acid in all groups. Significative quantitative differences (*p* < 0.0001) were observed for arginine, glutamine, and histidine content, which was higher in the CTRL group, and glycine was mainly abundant in TRT1. The PCA plot showed a separate clustering for chestnut-supplemented larvae meals compared to CTRL in terms of the free amino acid profile (Figure 4).

### 3.5. Bacterial Inhibition Activity of Tenebrio molitor Larvae Meal

**Figure 5:**
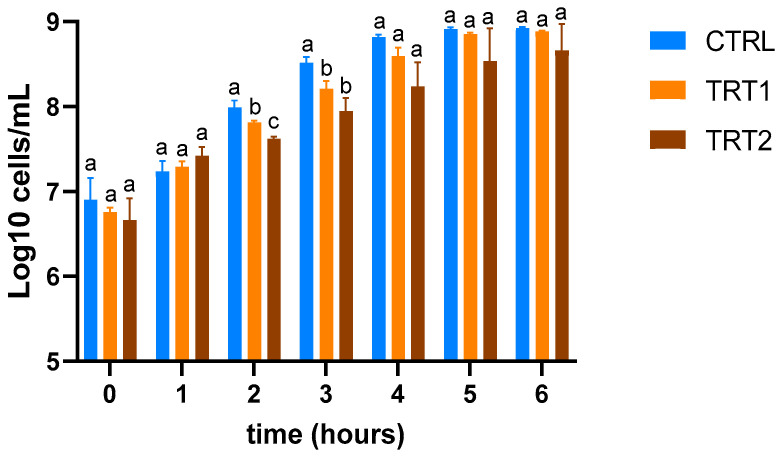
Evaluation of growth inhibition of *E. coli* O138 of insect meals in the control (CTRL) and treatment groups fed supplementation of 12.5% chestnut shell (TRT1) and 25% chestnut shell (TRT2). Data were registered for six hours after the inoculation. Results are presented as means ± standard deviations. ^a,b,c^ Lowercase letters indicate statistically significant differences among tested groups (repeated measurements ANOVA; interaction effect: *p* = 0.0118; F = 2.487; DFn = 12, DFd = 52).

The insect meals obtained from TRT1 and TRT2 showed higher inhibitory effects compared to CTRL (Figure 5) at the second (T2) and third hours (T3) compared to the TRT2 (*p* < 0.05). At the second hour (T2), the highest growth inhibition effect was observed in TRT2 compared to the other two groups (*p* < 0.05).

## 4. Discussion

In this study, the experimental design focused on the last period of the larval stage to maximise the effect of innovative substrates on the composition of insect meals.

Although significant differences were registered in substrate consumption, the biological significance of this difference could be negligible (approximately a 1% increase). Significant differences were observed in total larval biomass or growth rate between the CTRL and TRT2 groups at the end of the trial. Furthermore, FCR was significantly different in both periods, with higher efficiency in the CTRL group compared to TRT1 and TRT2 during the first 7 days. This trend reversed from days 7 to 14. Over the entire experimental period (treatment effect), FCR was similar among the groups, suggesting that the larvae likely adapted to the new substrate supplementation, resulting in comparable growth after 14 days. The increase in the survival rate of the larvae of TRT2 compared to the CTRL group suggests a positive effect of chestnut shell supplementation on the health of insects. Nevertheless, the effects appear to be dose-dependent because no differences were observed between TRT1 and CTRL in terms of weight gain and survival rate. The skin of the chestnut comprises two distinct layers: the outer pericarp and the inner integument [34]. Within these layers, lignin and carbohydrates emerge as the predominant components. Ramzy et al. [35] investigated the impact of a different level of lignin (up to 39%) on insect substrates: a negative correlation was observed between the increase in lignin content and the survival rate of larvae with an acceptable level up to 26% in the growth substrate. The bioactive molecules found in chestnut shell extracts play a crucial role in scavenging free radicals that protect cells from oxidative damage [36,37]. Natural antioxidants could activate mechanisms that stimulate the immune system of invertebrates. The oxidative stress could negatively influence insect growth; on the contrary, molecules of the chestnut shell could positively impact the survival and the weight gained by insects, which probably happened in this study. As observed in other species [38], the tannin content present in chestnut shells may also have positively influenced the health status of the larvae. Insects contain important levels of lipids; consequently, the antioxidant properties for lipid peroxidation inhibition revealed in chestnut skin [39] could potentially help protect the lipids in the insect’s cell membranes from damage caused by oxidative stress. A similar result was observed in a previous study that presented a similar experimental design, where *T. molitor* larvae were supplemented with lactoferrin as a hydration source [40]. The results demonstrated a protective effect of lactoferrin that raised the larvae survival rate over a 14 day trial, suggesting that compounds with antioxidant and antimicrobial properties can enhance the oxidative balance of insects. In our study, both treatments with chestnut shell supplemented in the growth substrate (TRT1 and TRT2) provided lower protein compared to the CTRL group; nevertheless, larvae of TRT1 and TRT2 achieved comparable performance. The higher survival rate that was registered contradicts the findings of [41], who reported a decrease in survival rates with lower protein in rearing substrates. Hence, our data supports the hypothesis that bioactive compounds present in chestnut shell may exert a positive influence on both larval growth and health. Therefore, although the definitive causative factors leading to the enhanced survival rates remain elusive, the available evidence strongly suggests that the chestnut shell may have exerted a positive influence on the insects’ survival. Further investigation is warranted to elucidate the specific mechanisms underlying this observed phenomenon.

Insects’ meal contains a high quantity of proteins, lipids, fatty acids, and micronutrients [42]. Considering the chemical composition of meals, our data showed higher protein content in TRT1 compared to CTRL and TRT2 insect meals, suggesting the capability of *T. molitor* to successfully bioconvert by-products with low-protein and high-fibre content into higher-value products. Our results are in line with the study of Fuso et al. [43] that observed a positive correlation between the protein content of insects and the fibre present in the growth substrate. In this study, the low percentage of proteins provided to the larvae resulted in a final product with high-value content. Larvae of the TRT1 and TRT2 groups exhibited protein contents of 51.96 ± 6.89% (on a dry matter basis) and 46.22 ± 6.23% (on a dry matter basis), respectively. These data are in line with the protein values of *T. molitor* meals reported in a previous study (CP: 43–51% on DM) [44]. The assessment of crude protein content in insect meals involved a conversion factor for nitrogen of 4.76 in order to avoid an overestimation of the protein content due to chitin presence, as suggested by Janssen et al. [27]. Protein concentration was significantly lower in the meal of TRT2 compared to TRT1, probably due to a different metabolic activity influenced by the substrate composition. A growth substrate with a higher fibre content may lead to an energy allocation towards sustaining insects’ basic metabolic functions rather than investing in protein synthesis for growth [45].

In this study, we have evaluated the content of free amino acids in *T. molitor* larvae related to different growth substrates, observing peculiar qualitative and quantitative trends. For instance, a significative reduction in histidine, glutamine and arginine was observed in TRT1 and TRT2, whereas the opposite trend was observed for glycine. Moreover, it is noteworthy to underline that the free amino acid profile of *T. molitor* by means of NMR spectroscopy has been previously reported [46]. Due to the different quantitative approaches used, it is not possible to make a quantitative comparison between the two studies. Generally, the concentrations of amino acids in the TRT2 group were lower for leucine, valine, arginine, glycine, proline, lysine, glycine, tyrosine, phenylalanine, and histidine compared to the CTRL group. This could be mainly due to the lower content of total protein on a DM basis that reflected, in general, a lower concentration of different amino acids. However, the differences observed in TRT1 and CTRL were mostly related to a different total protein content and a shift in the insects’ metabolism. From a qualitative point of view, several metabolites were identified in the present study: methionine, aspartate, asparagine, lysine, serine, tryptophan, and pyroglutamate. Interestingly, the chestnut shell supplementation in the growth substrate produced a modification of free amino acid metabolism, resulting in two discriminating factors: the presence of pyroglutamate and the absence of asparagine, in TRT1 and TRT2 compared to CTRL. This data represents an important novelty in the understanding of insects’ metabolism and the effect of functional compound supplementation in the growth substrates. The presence of pyroglutamate could probably be related to a massive cyclisation of glutamine concentration in the TRT1 and TRT2 groups. The conversion process from glutamine into pyroglutamic acid could occur under different pH conditions (<2 and pH > 13) or high temperatures involving a massive cyclisation process that releases ammonia [47]. In our study, all groups were subjected to the same heating conditions but TRT1 and TRT2 presented lower levels of free glutamine and the presence of pyroglutamate as opposed to the CTRL group where the latter is absent. In addition, the NMR analysis does not seem to risk inducing the cyclisation of glutamine to pyroglutamic acid in situ, unlike commonly used methods for measuring amino acid concentration, such as mass spectrometry [48]. Interestingly, concerning the biological aspect, previous studies reported that pyroglutamic acid shows antimicrobial activity and this property could be correlated to the higher antibacterial inhibition activity observed in TRT1 and TRT2 insect meals [49,50]. According to the study of Ludwig et al. [51], the amino acid profile, particularly the levels of phenylalanine and tyrosine, is correlated with the pigmentation of insects, as their decreases are likely associated with cuticular pigmentation. Indeed, it has been observed that the xylose and fucose in complex glycans, attached to asparagine, are strongly linked to the allergenicity of major glycoprotein allergens in plants and insects [52]. From a nutritional point of view, the insect meals in TRT1 presented comparable levels of lysine, methionine, and threonine to CTRL, which are the limiting amino acids for pigs’ feeding [53]. These data underline that supplementing the growth substrate with chestnut shell enriched the insects in terms of protein content and functional activity, without compromising the balance of essential amino acids for swine growth. This indicates promising suitability for further inclusion in monogastric diets. Due to the different free amino acid profiles, the insect meals of groups supplemented with chestnut shell in TRT1 and TRT2 were separately clustered within the PCA analysis. This suggests that these data could provide important insights for future regulations, particularly regarding laboratory analysis, product integrity, and the verification of rearing methods.

As the nutrient composition analyses proved to be significantly affected by diet, the colorimetric analysis of the insect meals revealed that substrates containing chestnut shells underlined differences in the colour of larvae. Insect meals from larvae fed with wheat bran displayed a lighter colour compared to those from insects that received chestnut shells. Even though Lee et al. [54] reported that a darker colour of insect meals in larvae reared on substrates presented low-quality protein, indicating a positive correlation between protein ingestion and cuticular melanisation, our results suggest a potential alternative explanation related to the presence of tannins, natural pigments found in chestnut shells and possibly bioaccumulated in larvae. These findings confirm that the colour of insect meals can be significantly affected by the substrate as also observed by Ferri et al. [40]. Stronger melanisation is known to enhance immune function by stimulating the prophenoloxidase system [55]. Therefore, our results suggest a positive correlation between the influence of chestnut shell and the melanisation of larvae cuticles. Functional ingredients in growth substrates may indirectly influence the expression of antimicrobial peptides [56]. This phenomenon could make insects more resilient to pathogens, thereby increasing their chances of survival. By incorporating bioactive ingredients into the rearing substrates, larvae have the potential to enhance the functional properties of insect meals, as highlighted by Andreadis et al. [57].

In this study, the supplementation of chestnut shell showed the increased capacity of insect meal extracts to inhibit *Escherichia coli* O138 growth, a representative model of gastrointestinal disorders [58]. Results from *E. coli* inhibitory activity have demonstrated that the TRT1 and TRT2 meal extracts, at concentrations of 1:4, inhibited the growth of *E. coli* O138 more than the CTRL group meal extract at different time points after the bacterial inoculation. It has been recognised that insects have a natural antibacterial activity: the capability of inhibiting bacterial growth may be modulated by the bioactive molecules of larvae such as chitin and AMP. Patyra and Kwiatek [17] suggest the use of AMP from insects against stressful situations in livestock, such as the transport of animals or changes in temperature, which are usually associated with a reduction in immune defences and increased risk of infection. Previous studies have confirmed the antimicrobial activity of insect meals in animal nutrition with encouraging results in animal performance, nutrient digestibility, and intestinal morphology. In our trial, the presence of tannins in chestnut shells may have increased the natural inhibitory activity of mealworms and, consequently, of meal. An in vivo study on weaned piglets showed a positive effect of insect AMPs on reducing the incidence of diarrhoea at 15 through 28 days of the trial in animals fed with feed supplemented with 5% *T. molitor* [59]. Meyer et al. [60] demonstrated that the inclusion of *T. molitor* larvae meal containing 0.5% and 1% chitin in the diet of growing pigs could modulate caecal microbiota composition. This inhibitory activity can contribute to promoting animal health and reducing the need for antibiotics, a topic of great interest in animal production to counteract the phenomenon of antibiotic resistance.

An important debate has arisen regarding whether the chitin component of insects can negatively affect nutrient digestibility through the encapsulation of nutrients [61]. Nevertheless, our in vitro digestibility values of *T. molitor* meals were not significantly different among groups (range 57–61%); these results are consistent with data reported in the literature and are comparable to the digestibility of other protein sources used in animal nutrition, such as soybean meal [62]. Moreover, in vivo digestibility could be higher compared to in vitro digestibility. Fanimo et al. [63] reported that chitinolytic enzymes, including chitinase and chitobiase, are responsible for chitin digestion in pigs. These enzymes are produced in considerable amounts by microorganisms in the digestive tract of several mammalian species [64].

## 5. Conclusions

In conclusion, our study highlighted that including chestnut shells in the insect growth substrate enhances larval survival rates and improves the nutritional profile of *T. molitor* larvae meal, which showed higher protein content, a modulated amino acid profile and different lipid levels despite lower initial concentrations in the growth substate. This highlights the potential of valorising by-products in insect rearing to enhance both survival and nutritional quality. Further investigations are needed to elucidate the metabolic mechanisms of *T. molitor* larvae and the broader implications of enriched insect meals for functional nutrition.

## Figures and Tables

**Table 1:** Metabolites and relative ^1^H NMR signals selected for quantitative analysis in the Bligh-Dyer hydroalcoholic extracts.

Metabolite	Chemical Shift (ppm)
Leucine	0.97
Isoleucine	1.02
Valine	1.05
Threonine	1.34
Alanine	1.48
Arginine	1.66
Proline	2.02
Glutamine	2.46
Methionine	2.65
Aspartate	2.81
Asparagine	2.88
Lysine	3.01
Betaine	3.27
Glycine	3.57
Serine	3.82
Pyroglutamate	4.19
Tyrosine	6.90
Histidine	7.12
Phenylalanine	7.43
Tryptophan	7.55

**Table 2:** Growth performance of insects in the control (CTRL), and treatment groups where larvae received a supplementation of 12.5% chestnut shell (TRT1) and 25% chestnut shell (TRT2).

Item	CTRL	TRT1	TRT2	*p*-Values				
				Trt	Time	TrtxTime	F-Value	DF (n, d)
Substrate consumed (g)				0.0119	0.5931	0.4485	0.83	2, 21
d 0–7	96.98 ± 0.35 ^a^	96.75 ± 0.12 ^b^	96.72 ± 0.23 ^b^					
d 7–14	96.87 ± 0.08	96.79 ± 0.04	96.70 ± 0.04					
Average weight (g)				0.1453	<0.0001	0.0030	4.74	4, 42
d 0–7	104.5 ± 4.07	102.4 ± 2.05	104.43 ± 2.16					
d 7–14	104.9 ± 7.60	109.4 ± 3.37	112.3 ± 2.93					
Growth rate (%)				0.1658	0.0018	0.0280	4.34	2, 19
d 0–7	4.47 ± 4.09	2.41 ± 2.50	4.33 ± 2.17					
d 7–14	4.10 ± 0.41 ^a^	6.80 ± 1.48 ^ab^	7.60 ± 1.54 ^b^					
FCR				0.5344	0.0196	0.0015	12.48	2, 11
d 0–7	7.91 ± 2.22 ^a^	14.61 ± 3.28 ^b^	12.58 ± 2.52 ^b^					
d 7–14	11.79 ± 1.45 ^a^	8.11 ± 1.64 ^ab^	7.89 ± 1.46 ^b^					

All values are presented as means ± standard deviations. FCR = feed conversion ratio as percentages on dry matter bases presented as means ± standard deviations. Means within a row with different lowercase letters are significantly different (*p* < 0.05).

**Table 3:** Chemical analysis of growth substrates administered to insects in the control (CTRL) and treatment groups where larvae received a supplementation of 12.5% chestnut shell (TRT1) and 25% chestnut shell (TRT2).

Components (%)	CTRL	TRT1	TRT2	*p*-Value	F-Value/ Kruskal–Wallis	DF (n, d)
Dry matter	90.62 ± 1.26	91.89 ± 1.39	91.63 ± 1.35	0.5094	0.76	2, 6
Ash	6.09 ± 1.86	7.98 ± 1.65	6.55 ± 0.63	0.3393	2.49	-
Ether Extract	2.93 ± 0.94	1.65 ± 0.05	1.73 ± 0.20	0.0523	5.02	2, 6
Crude Fibre	11.87 ± 4.30	12.26 ± 0.97	14.85 ± 0.43	0.3647	1.20	2, 6
Crude Protein	17.00 ± 0.42 ^a^	14.48 ± 0.20 ^b^	15.09 ± 0.27 ^b^	0.0001	53.68	2, 6
Non-Structural Carbohydrates	62.11 ± 3.77	63.62 ± 2.14	61.78 ± 0.66	0.6572	0.45	2, 6

All values are expressed as percentages on dry matter bases and are presented as means ± standard deviations. Means within a row with different lowercase letters are significantly different (ANOVA and Kruskal–Wallis tests, *p* < 0.05).

**Table 4:** Nutritional composition of insect meals in the larvae fed wheat bran (CTRL), and larvae fed wheat bran supplemented with 12.5% and 25% chestnut shell (TRT1 and TRT2).

Components	CTRL	TRT1	TRT2	*p* Value	F-Value/Kruskal–Wallis	DF (n, d)
Dry matter	93.3 ± 3.35 ^a^	81.45 ± 7.64 ^b^	82.87 ± 2.24 ^b^	0.0002	13.50	2, 21
Crude protein (%)	44.52 ± 2.96 ^a^	51.96 ± 6.89 ^b^	46.22 ± 6.23 ^a^	0.0391	6.49	-
Ether Extract (%)	31.14 ± 2.79 ^a^	36.09 ± 4.40 ^b^	32.51 ± 1.21 ^ab^	0.0123	5.47	2, 21
Ash (%)	5.01 ± 0.36 ^ab^	3.76 ± 0.14 ^a^	8.01 ± 0.30 ^b^	<0.0001	16.80	-

All values are expressed as percentages on a dry matter basis and are listed as means ± standard deviations. Means within a row with different letters are significantly different (ANOVA and Kruskall–Wallis tests, *p* < 0.05).

**Table 5:** Lightness (L*), red/green (a*) and yellow/blue (b*) values of the control group (CTRL) and insect meals from larvae fed chestnut shells at 12.5% (TRT1) and chestnut shells at 25% (TRT2).

Insect Meals		L*	a*	b*
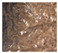	CTRL	33.07 ± 1.32 ^a^	6.19 ± 0.49	13.68 ± 0.87 ^a^
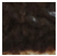	TRT1	27.96 ± 2.87 ^b^	6.68 ± 0.43	11.56 ± 1.27 ^b^
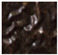	TRT2	30.20 ± 1.66 ^ab^	6.64 ± 0.33	12.01 ± 0.78 ^ab^
	*p* value	0.0021	0.0731	0.0072
	Kruskal–Wallis	12.29	5.23	9.88

All values are expressed as means ± standard deviations. Means within a column with different letters are significantly different (Kruskall–Wallis, *p* < 0.05).

**Table 6:** Amino acid content (mg/100 g of insect meals on a dry weight basis) of *Tenebrio molitor* meals of different growth substrates: wheat bran (CTRL), wheat bran with 12.5 and 25% chestnut shell (TRT1 and TRT2).

Amino Acids and Derivatives	Group					
	CTRL	TRT1	TRT2	*p*-Value	F-Value/Kruskal–Wallis	DF (n, d)
Leu	56.56 ± 5.48 ^a^	57.99 ± 8.92 ^a^	46.62 ± 4.98 ^b^	0.0007	11.59	-
Ile	69.12 ± 4.49	65.58 ± 4.93	65.67 ± 8.45	0.5142	0.69	2, 18
Val	143.6 ± 8.65 ^a^	118.0 ± 5.64 ^b^	121.5 ± 11.22 ^b^	0.0001	15.90	2, 18
Thr	36.00 ± 5.80	48.26 ± 12.31	39.88 ± 8.12	0.0972	4.621	-
Ala	57.92 ± 6.72 ^a^	83.45 ± 17.71 ^b^	62.06 ± 7.87 ^a^	0.0015	9.49	2, 18
Arg	399.0 ± 34.25 ^a^	273.2 ± 23.63 ^b^	299.7 ± 52.38 ^b^	<0.0001	18.46	-
Gln	126.4 ± 40.34 ^a^	16.04 ± 4.38 ^b^	25.99 ± 6.85 ^b^	<0.0001	16.10	-
Pro	966.8 ± 39.39 ^a^	880.7 ± 52.54 ^b^	892.7 ± 77.80 ^ab^	0.0342	4.10	2, 18
Met	5.31 ± 1.09	5.69 ± 2.18	3.51 ± 1.64	0.0389	6.20	-
Asp	18.76 ± 2.15 ^a^	27.39 ± 6.35 ^b^	20.84 ± 2.53 ^ab^	0.0034	9.81	-
Asn	6.31 ± 2.29	n.d.	n.d.	-	-	-
Lys	170.4 ± 16.00 ^a^	171.9 ± 36.93 ^a^	124.6 ± 10.46 ^b^	0.0005	12.09	-
Gly	65.81 ± 15.80 ^a^	142.8 ± 31.07 ^b^	90.29 ± 17.97 ^a^	<0.0001	15.64	-
Tyr	186.9 ± 25.63 ^a^	161.3 ± 17.94 ^ab^	152.8 ± 17.27 ^b^	0.0218	7.13	-
Phe	31.83 ± 3.92 ^a^	30.16 ± 3.42 ^a^	22.77 ± 3.06 ^b^	0.0002	12.95	-
Trp	61.85 ± 5.62 ^ab^	84.62 ± 32.38 ^a^	53.80 ± 3.51 ^b^	0.0039	9.66	-
His	146.6 ± 12.63 ^a^	95.30 ± 9.24 ^b^	107.3 ± 12.33 ^b^	<0.0001	13.75	-
Ser	56.54 ± 5.48 ^a^	21.76 ± 4.15 ^b^	45.50 ± 18.53 ^a^	0.0018	10.59	-
PyroGlu	n.d.	223.5 ± 25.56	206.0 ± 39.46	0.3665	2.38	7, 5

All values are expressed as mg per 100 g of insect meals on a dry weight basis and are presented as means ± standard deviations. Means within a row with different letters are significantly different (ANOVA; unpaired Student’s *t*-test; *p* < 0.05). n.d.: not detected.; Leu: leucine; Ile: isoleucine; Val: valine; Thr: threonine; Ala: alanine; Arg: arginine; Gln: glutamine; Pro: proline; Met: methionine; Asp: aspartate; Asn: Asparagine; Lys: lysine; Gly: glycine; PyroGlu: pyroglutamate; Tyr: tyrosine; Phe: phenylalanine; Trp: tryptophan; His: histidine; Ser: serine.

## Data Availability

All data are available within the manuscript and from the corresponding author upon reasonable request.
